# Phytochemical analysis, in-vitro anti-proliferative, anti-oxidant, anti-diabetic, and anti-obesity activities of *Rumex rothschildianus* Aarons. extracts

**DOI:** 10.1186/s12906-021-03282-6

**Published:** 2021-03-31

**Authors:** Nidal Jaradat, Mohammed Hawash, Gada Dass

**Affiliations:** Department of Pharmacy, Faculty of Medicine and Health Sciences, An-Najah National University, Nablus, 00970 Palestine

**Keywords:** *Rumex rothschildianus*, Antioxidant, Lipase, Amylase, Trolox, Phytochemistry, Anti-proliferative activity

## Abstract

**Background:**

*Rumex rothschildianus* is the sole member of a unique section of the genus *Rumex,* in the family Polygonaceae. This species is a very rare small dioecious annual, endemic to Palestine that is traditionally used as food and for the treatment of various diseases. Therefore, the current investigation aimed to screen the chemical constituents, antioxidants, anti-α-amylase, anti-α-glucosidase, antilipase, and cytotoxic effects of four solvents fractions of *R. rothschildianus* leaves.

**Methods:**

Dried powder of *R. rothschildianus* leaves was extracted in four solvents with different polarities. Several qualitative and quantitative phytochemical tests were performed to determine the components of the extracts. The colorimetric analysis was used for the quantitative determination of phenols, flavonoids, and tannins. In-vitro assays were performed to evaluate the extracts for antioxidant, anti-α-amylase, anti-α-glucosidase, and antilipase inhibitory activities, as well as cytotoxicity by MTS assay against cervical carcinoma cells line (HeLa) and breast cancer cell line (MCF7).

**Results:**

The acetone fraction of *R. rothschildianus* leaves showed the most significant antioxidant activity, due to having the highest content of flavonoids and phenolics, with an IC_50_ value of 6.3 ± 0.4 μg/ml, compared to 3.1 ± 0.9 μg/ml for Trolox, and regarding lipase inhibition activity the acetone fraction showed the most potent activity with an IC_50_ value of 26.3 ± 0.6 μg/ml, in comparison with orlistat positive control IC_50_ 12.3 μg/ml. The same extract was the most potent inhibitor of α-amylase and α-glucosidase, with IC_50_ values of 19.1 ± 0.7 μg/ml and 54.9 ± 0.3 μg/ml, respectively, compared to 28.8, 37.1 ± 0.3 μg/ml of acarbose, respectively. The hexane fraction showed 99.9% inhibition of HeLa cells and 97.4% inhibition for MCF7 cells.

**Conclusion:**

The acetone fraction of *R. rothschildianus* leaves might provide a source of bioactive compounds for the treatment of oxidative stress. Similarly, the hexane fraction indicates the promising antitumor potential of *R. rothschildianus*. Clearly, these initial indications need further purification of potentially active compounds, and ultimately, in-vivo studies to determine their effectiveness.

## Background

Plants have been used as therapies since ancient times. Roots, seeds, bark, leaves, and flowers have all been used for remedial purposes. In the present day, synthetic medicines are available and are effective in the treatment of a wide range of diseases; however, some people still prefer herbal medicines as they are viewed as being less harmful to the human body [1, 2]. Medicinal plants are by definition the source of phytochemical compounds that possess therapeutic activities. These properties rely upon the presence of different secondary metabolites, such as phenolic, terpenoids, and alkaloids [3].

*Rumex rothschildianus* Aarons*.* is the sole member of a unique section of the genus Rumex, in the family Polygonaceae. This species is a very rare small dioeciously annual, endemic in Palestine. It has a mean height of 45 cm, is characterized by erect stems holding radical petiolate leaves, which are short-hastate at the base and short-acuminate at the apex. Flowers have a diameter of 3–4 mm, while pistillate flowers are about 2 mm in diameter with a coriaceous membranous layer [4]. *Rumex spp.* are widespread in different regions of Turkey, are represented by 22 species. Some of the most common species are *R. patientia* L.*, R. crispus* L*., R. acetosa* L*. R. caucasicus rech.,* and *R. alpinus* L. *R. alpinus* and *R. caucasicus* are perennial plants distributed in middle and eastern Anatolia at an altitude of 1000–3000 m above sea level. The Rumex genus has been widely used in traditional medicine in Turkey to treat disorders, such as constipation, diarrhea, and eczema [5, 6]. The genus also has some laxative, diuretic, antipyretic, wound healing, and anti-inflammatory effects. Many people in the eastern part of Turkey use young leaves of Rumex spp. as a preservative in cheese, as well as giving food aroma [7].

A variety of research has been carried out on Rumex species, such as antimicrobial activities being reported for some species. Some bioactive phytochemicals have previously been found in *Rumex vesicarius* L*.,* such as carotenoids, tocopherols, polyphenols, flavonoids, and ascorbic acid, which have a role as antioxidants and natural detoxifying agents. The dietary intake of antioxidant phytochemicals, like carotenoids, phenolics, and flavonoids may protect against non-communicable diseases in humans, such as cancer, cardiovascular disorders, and other health problems related to oxidative stress [5, 8].

Harmful free radicals are known to play an important role in many major health problems, such as cancer, cardiovascular disease, rheumatoid arthritis, cataracts, and Alzheimer’s disease, and other degenerative diseases related to aging. Antioxidants are beneficial components that neutralize these free radicals before they can attack cells, and hence they prevent damage to cell proteins, lipids, and carbohydrates. A variety of both natural and synthetic antioxidants have been proposed for the treatment of human diseases. Such interest in the role of antioxidants in human health has prompted research in the fields of food science and medicinal herbs, assessing the function of herbs as antioxidants. Antioxidant action includes free radical scavenging capacity, inhibition of lipid per̴oxidation, metal ion chelating ability and also reducing capacity [9, 10].

Cancer is one of the most global health care problems. The development and discovery of novel anticancer medication remain extremely important due to various factors. These factors include treatments that may cause major side effects or can be rather expensive. Alternatives that are safer biologically and more affordable are still highly desirable [11–14].

Several plant species are considered potential sources of bioactive molecules such as atropine from Belladonna leaves, cocaine from coca leaves, vincristine from Vinca plant, and many others which still play an important role in modern medicine [15, 16].

Useful therapeutic effects can come from mixing secondary products present in medicinal plants. These compounds are mostly secondary metabolites, like alkaloids, steroids, tannins, flavonoids, and phenolic, which are synthesized and deposited in specific parts of these plants [17, 18]. The present study investigates the in-vitro anti-α-amylase, anti-α-glucosidase, anti-lipase, antiproliferative and antioxidant activities of different fractions extracted from *R. rothschildianus* leaves.

## Methods

### Plant material, chemicals, and instruments

*R. rothschildianus* leaves were harvested from Western regions of Palestine, between February and March 2018. They were identified by Dr. Nidal Jaradat, from the Pharmacognosy Laboratory at An-Najah National University, under the voucher specimen code Pharm-PCT-2066. All chemicals were purchased from Sigma-Aldrich. A spectrophotometer-UV/Visible (Jenway® 7135, Staffordshire, UK), filter papers (Whitman No. 1, Washington, USA), shaker device (Memmert 531–25-1, Stockholm, Sweden), rotavap apparatus (Heidolph-VV 2000, Schwabach, Germany), grinder (Aero Plus 500 W Mixer Grinder, I01, Wan Chai, China), electronic-balance (Radwag, AS 220/c/2, Toruńska, Poland), freeze dryer - BT85 (Millrock Technology, China) and cryo-desiccator (Mill-rock technology, BT85, Kingston, USA) were used.

### Preparation of extracts and fractionation

Dried powder of *R. rothschildianus* leaves was extracted by adding solvents sequentially based on their polarity, beginning with the non-polar solvent hexane, and then acetone (a polar aprotic organic solvent), methanol (polar alcohol), and finally distilled water (a polar protic solvent). For each extraction, about 25 g ground dried leaves were placed in 0.5 l hexane for 72 h in a shaker device at 100 rotations per minute at 25 °C. Firstly, the hexane was replaced with 0.5 L acetone, and then subsequently replacement involved equivalent volumes of methanol and water. Incubations in the solvents were as described above for hexane. Each organic fraction was filtered and concentrated under a vacuum on a rotary evaporator, while the aqueous fraction was dried using a freeze dryer. Finally, all crude fractions were stored at 4 °C [19, 20].

The yield of each fraction was calculated using the following formula:

$$ \mathrm{Yield}\%=\left(\mathrm{weight}\ \mathrm{of}\kern0.5em Rumex\kern0.5em \mathrm{extract}/\mathrm{original}\ \mathrm{dry}\ \mathrm{weight}\ \mathrm{of}\kern0.5em Rumex\kern0.5em \mathrm{leaf}\ \mathrm{tissue}\right)\times 100\% $$

### Preliminary phytochemical assessment

Phytochemical screening tests of *R. rothschildianus* leaves four fractions were carried out to identify active secondary metabolites. The qualitative results were expressed as (+) for the presence and (−) for the absence of bioactive phytochemicals [10, 21].

### Determination of total phenolic content (TPC)

The procedure to determine TPC was based on that of Cheung et al. TPC was expressed in milligram of gallic acid equivalents per gram dry weight of leaves (mg GA/g dry weight). Freshly prepared 7.5% sodium carbonate solution was made by placing 7.5 g Na_2_CO_3_ in a volumetric flask and adjusting the volume to 100 ml with distilled water. A standard reference solution (gallic acid solution) was prepared by dissolving 100 mg of gallic acid in distilled water to a final volume of 100 ml. From this, a serial dilution was performed to obtain solutions of gallic acid at 100, 70, 50, 40, and 10 μg/ml). The stock solutions of the fractions from leaves were prepared by dissolving 100 mg plant extract in distilled water, adjusted to a total volume of 100 ml. Reaction mixtures were prepared by mixing 0.5 ml of each fraction solution with 2.5 ml 10% Folin-Ciocalteu’s reagent, which was dissolved in water with 2.5 ml 7.5% sodium bicarbonate. The sample tubes were incubated for 45 min at 45 °C. Then, the absorbance of each was measured in a spectrophotometer at wavelength 765 nm. The working samples were prepared in triplicate for each analytic trial, from which the mean and standard deviation values were calculated [21].

### Determination of total flavonoid content (TFC)

The TFC in the four *R. rothschildianus* leaf fractions was assessed using a calibration curve of rutin (standard reference compound). Results were expressed as milligram of rutin equivalent per gram dry weight of leaves extract (mg RU/g dry weight). A calibration curve for rutin was established using serial dilutions generated from a stock solution of 100 μg/ml. To make the stock solution, 10 mg of rutin was dissolved in 10 ml of distilled water and then diluted to 100 ml. Subsequently, the stock solution was diluted to provide rutin at concentrations of 10, 30, 40, 50, 70, and 100 μg/ml. For working solution preparation, 0.5 ml of each fraction solution was mixed with 3 ml methanol, 0.2 ml 10% AlCl_3_, 0.2 ml 1 M potassium acetate and 5 ml distilled water, and then incubated at room temperature for 30 min. The previous steps were repeated for each of the fractions, after which, absorbance was measured at a wavelength of 415 nm. For a blank control, a working solution was set up with distilled water in place of the sample extract. The samples were prepared in triplicate for each analytic trial, from which the mean and standard deviation values were calculated [22].

### Determination of total tannin content (TTC)

The protocol of Sun et al. was used to determine TTC in the four *R. rothschildianus* leaf fractions, being the most commonly used procedure. Catechin was used as a reference compound to construct a calibration curve. A 100 μg/ml stock in methanol was prepared, from which a dilution series was generated to give catechin concentrations of 10, 30, 50, 70, and 100 μg/ml. A 4% solution of vanillin in methanol was prepared freshly. Stock solutions of the fractions at 100 μg/ml were prepared using methanol as a solvent. For the working solution, 0.5 ml of each fraction solution was mixed with 3 ml vanillin solution and 1.5 ml of concentrated HCl. The mixture was allowed to stand for 15 min, and then the absorbance at 500 nm was measured, using a working solution set up with methanol in place of the sample extract as a blank. All working samples were analyzed in triplicate, from which the mean and standard deviation values were calculated. Total tannin in each fraction was expressed in terms of catechin equivalents (mg of CAE/g dry weight of leaves) [23].

### Antioxidant activity method

The free 2,2-diphenyl-picrylhydrazyl (DPPH) radical scavenging assay was used to measure antioxidant activity in the different fractions of *R. rothschildianus* leaves. A 1000 μg/ml stock solution of each plant fraction was prepared in methanol. In addition, a 1000 μg/ml solution of trolox was also prepared (the reference standard). A dilution series was prepared from the stock solutions for each fraction, giving six serial dilutions at 2, 5, 10, 20, 50, and 100 μg/ml. One ml of each extract dilution was mixed with 1 ml 0.002 g/ml DPPH in methanol. One ml methanol was added to give a final working volume of 3 ml. The DPPH solution was freshly prepared, as it was very sensitive to light. The blank control of the series concentrations was DPPH in methanol in a ratio of 1:2, without the addition of an extract. All working solutions were incubated at room temperature (25 °C) in the dark for about 30 min. Optical densities were then measured with a spectrophotometer at a wavelength of 517 nm. The following equation was used to calculate % DPPH inhibition for each plant fraction, with trolox as the standard compound:

$$ \mathrm{DPPH}\ \mathrm{inhibition}\%=\left({\mathrm{A}}_{\mathrm{B}}-{\mathrm{A}}_{\mathrm{ts}}\right)/{\mathrm{A}}_{\mathrm{B}}\times 100\% $$

where, A_B_ is the recorded absorbance of the blank solution, and A_ts_ is the recorded absorbance of the tested sample solution [21].

### Porcine pancreatic lipase inhibition assay

Stock solutions of 500 μg/ml were made from each plant fraction in 10% DMSO. From these, a dilution series of five concentrations of 50, 100, 200, 300, and 400 μg/ml were made. A 1 mg/ml stock solution of porcine pancreatic lipase in Tris-HCl buffer was prepared freshly just before use. The substrate, p-nitrophenyl butyrate (PNPB) was prepared by dissolving 20.9 mg in 2 ml acetonitrile.

For each working solution, 0.1 ml porcine pancreatic lipase was mixed with 0.2 ml plant fraction from each member of the dilution series. Tris-HCl was added to make the final volume of the working solutions 1 ml, and they were incubated at 37 °C for 15 min. After incubation, 0.1 ml p-nitrophenyl butyrate solution was added to each test-tube. The mixture was then incubated for a further 30 min at 37 °C. Pancreatic lipase activity was determined by measuring the hydrolysis of PNPB into p-nitrophenolate at 410 nm, using a UV spectrophotometer. The same procedure was repeated using orlistat as a standard reference compound. Percentage lipase inhibition by plant fractions was calculated with the following equation:

$$ \mathrm{Lipase}\ \mathrm{inhibition}\%=\left({\mathrm{A}}_{\mathrm{B}}-{\mathrm{A}}_{\mathrm{ts}}\right)/{\mathrm{A}}_{\mathrm{B}}\times 100\% $$

where, A_B_ is the recorded absorbance of the blank solution, and A_ts_ is the recorded absorbance of the tested sample solution [24].

### α-Amylase inhibitory assay

A100 mg of each fraction was dissolved in a few milliliters of 10% DMSO, and then further dissolved up to 100 ml in 0.02 M Na_2_HPO_4_/NaH_2_PO_4_, 0.006 M NaCl, pH 6.9 to give finally stock solutions with concentrations of 1000 μg/ml. From these, the following dilutions were prepared of 10, 50, 70, 100, and 500 μg/ml, using 10% DMSO as the diluent. A 0.2 ml volume of 2 units/ml porcine pancreatic α-amylase was mixed with 0.2 ml plant fraction and was incubated for 10 min at 30 °C. After incubation, 0.2 ml of a freshly prepared 1% starch solution in water was added, and the tubes were then incubated for at least three more minutes. At this point, the reaction was stopped by the addition of 0.2 ml 3,5-dinitro salicylic acid (DNSA) color reagent and was diluted with 5 ml of distilled water, before being heated at 90 °C for 10 min in a water bath. The mixture was then cooled to room temperature, and the absorbance was measured at 540 nm. The blank control was prepared using the same quantities described above, but replacing the plant fraction with 0.2 ml buffer. Acarbose was used as a standard reference following the procedure described above. α-amylase inhibitory activity was calculated using the following equation:

$$ \%\mathrm{of}\ \alpha -\mathrm{amylase}\ \mathrm{inhibition}=\left({\mathrm{A}}_{\mathrm{B}}-{\mathrm{A}}_{\mathrm{T}}\right)/{\mathrm{A}}_{\mathrm{B}}\times 100\%, $$

where, A_B_: is the absorbance of the blank sample, and A_T_ is the absorbance of the test sample [25].

### α-Glucosidase inhibitory assay

A dilution series of fractions was made, to yield concentrations of 100, 200, 300, 400, and 500 mg/ml. The reaction mixtures contained 0.1 ml 1 U/ml α-glucosidase solution mixed with 0.2 ml of an extract dilution and 0.5 ml 100 mM phosphate buffer, pH 6. 8. The mixtures were incubated at 37 °C for 15 min. Then, 0.2 ml 5 mM *p*-nitrophenyl α-D-galactopyranoside PNPG was added to the reaction mixture and incubated was extended for a further 20 min at 37 °C. The reaction was terminated by adding 0.1 M Na_2_CO_3_. The absorbance was recorded at a wavelength of 405 nm for all samples. Acarbose was used as a positive control at the same concentrations as the plant extracts. The results were expressed as percentage inhibition according to the following equation:

$$ \alpha -\mathrm{Glucosidase}\ \mathrm{Inhibition}\ \left(\%\right)=\left({\mathrm{A}}_{\mathrm{B}}-{\mathrm{A}}_{\mathrm{S}}/{\mathrm{A}}_{\mathrm{B}}\right)\times 100\% $$

where, A_B_ is the absorbance without enzyme inhibitor, and A_S_ is the absorbance in the presence of the enzyme inhibitor [26].

### Cell lines and MTS assay

HeLa and MCF7 cancer cell lines were obtained from ATCC, the cells were cultured in RPMI-1640 media supplemented with 10% fetal bovine serum, 1% penicillin/streptomycin antibiotics, and 1% L-glutamine. Cells were grown in a humidified atmosphere with 5% CO_2_ at 37 °C. Cells were seeded at 2.6 × 10^4^ cells/well in a 96-well plate. After 48 h, cells were confluent, the media was changed. Cells were then incubated with different leaf fraction concentrations ranging from 0.25–10 mg/ml of *R. rothschildianus* for 24 h. Cell viability was assessed with the CellTilter 96® Aqueous One Solution Cell Proliferation (MTS) Assay according to the manufacturer’s instructions (Promega Corporation, Madison, WI). Briefly, at the end of the treatment, 20 μl MTS solution per 100 μl media was added to each well and incubated at 37 °C for 2 h. Absorbances were measured at 490 nm [27, 28].

### Statistical analysis

All of the obtained results of the four studied plant fractions (antioxidant, anti-lipase, anti-amylase, anti-glycosidase, and cytotoxicity activities) were expressed as mean ± SD standard deviation; the result was considered significant when the *p-*value was < 0.05. Data were compared using unpaired *t*-tests.

## Results

### Phytochemical screening

The results of the preliminary phytochemical tests on the *R. rothschildianus* aqueous fractions showed the presence of saponin, phenols, protein, starch, and flavonoids. The methanol extract showed the presence of complex polysaccharides, phenols, protein, and flavonoids, while phenols, tannins, and flavonoids were observed in the acetone fraction, and in hexane fraction phenols, tannins, and flavonoids, glycosides, terpenoids, and steroids were identified as shown in Table 1. However, for the extraction process, methanol showed the highest percentage yield at 29.4%, followed by the acetone fraction with 16.5%. The aqueous extraction yielded 10.6%, while the lowest yield (7.3%) was in hexane fraction (Table 2).

**Table 1:** Phytochemical screening assessment of *R. rothschildianus* leaves four solvents fractions

Phytochemically active constituent	Hexane extract	Acetone extract	Methanol extract	Aqueous extract
Protein	–	–	+	++
Reducing sugars	–	–	–	–
Complex polysaccharides	–	–	+	–
Phenols	++	+++	+	+
Starch	–	–	–	+++
Tannins	+	+	–	–
Flavonoids	+++	+++	++	+
Saponins	–	–	–	++
Glycosides	+	–	–	–
Terpenoids and steroids	+	–	–	–
Alkaloids	–	–	–	–
Volatile oil	–	–	–	–

+: low content; ++ moderate content; +++ high content; − absent

**Table 2:** The percentage yield of *R. rothschildianus* leave’s fractions

Fraction	Extract (g)	Plant material (g)	Yield
Hexane	1.82	25	7.28%
Acetone	4.12	25	16.48%
Methanol	7.35	25	29.40%
Aqueous	2.66	25	10.64%

### Quantitative analysis of TPC, TFC, and TTC

For the evaluation of TPC, TFC, and TTC, the absorption (Abs) values of several concentrations of the gallic acid, rutin acid, and catechin standards (STDs) were obtained, and regarding these points, three equations were obtained for each STD versus its concentrations to calculate the total phenol, flavonoid, and tannin contents of the hexane, acetone, methanol, and aqueous *R. rothschildianus* fractions are presented in Table 3.

**Table 3:** Quantitation of phenols, tannins, and flavonoids in hexane, acetone, methanol, and aqueous fractions of *R. rothschildianus* leaves

Leaf Fractions	Total flavonoids content (TFC), mg of RUE/g leaf dry weight, ± SD	Total phenol content (TPC), mg of GAE/g leaf dry weight, ± SD	Total Tannin content (TTC), mg of CAE/g leaf dry weight, ± SD
Hexane	92.35 ± 2.33	17.66 ± 1.56	2.21 ± 0.01
Acetone	107.30 ± 4.60	28.20 ± 0.78	4.95 ± 0.77
Methanol	55.65 ± 2.33	5.44 ± 1.56	–
Aqueous	32.30 ± 2.35	1.89 ± 1.25	–

### Antioxidant activity

**Fig. 1:**
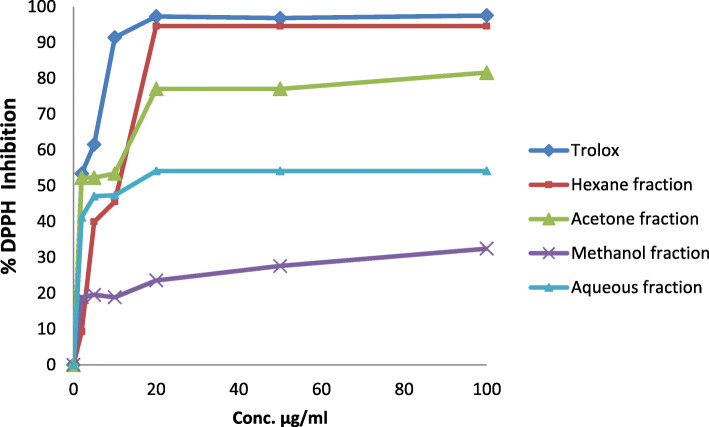
% Inhibition of DPPH for standard trolox and fractions from *R. rothschildianus* leaves

The results of assessing the free radical scavenging activity of four fractions from *R. rothschildianus* leaves, using trolox as a potent antioxidant standard reference, were expressed as percentage DPPH inhibition (Fig. 1 and Table 4). Therefore, *R. rothschildianus* leaves could be considered an herbal source for antioxidants, and for the acetone fraction, which showed an IC_50_ value of 6.3 ± 0.4 μg/ml. Similar results were also obtained for the hexane fraction, which had an IC_50_ value of 7.9 ± 1.3 μg/ml (Table 4). The results were compared to trolox, a potent antioxidant compound, with an IC_50_ equal to 3.1 ± 0.9 μg/ml. By contrast, the aqueous fraction only showed moderate antioxidant activity, with a higher IC_50_ value of 19.9 ± 0.7 μg/ml, while the methanol extract was inactive in this assay.

**Table 4:** The IC_50_ for different extracts fractions against DPPH, Lipase, α-Amylase and α-glucosidase in comparison of IC_50_ of positive controls

	Target enzyme	Reference	Hexane fraction	Acetone fraction	Methanol fraction	Aqueous fraction
IC_50_ (μg/mL)	**DPPH**	3.10 ± 0.92^a^	7.90 ± 1.32	6.30 ± 0.43	NI	19.95 ± 0.71
	**Lipase**	12.30 ± 0.33^b^	39.81 ± 0.27	26.30 ± 0.57	60.26 ± 0.42	NI
	**α-Amylase**	28.84 ± 1.22^c^	354.8 ± 1.17	19.05 ± 0.75	NI	45.70 ± 0.26
	**α-Glucosidase**	37.15 ± 0.33^C^	NI	54.90 ± 0.33	251.18 ± 0.43	NI

^a^ Trolox, ^b^ Orlistat, ^c^ Acarbose, NI: no inhibition (inhibition at conc. higher than 400 μg/ml)

### Lipase inhibition activity

**Fig. 2:**
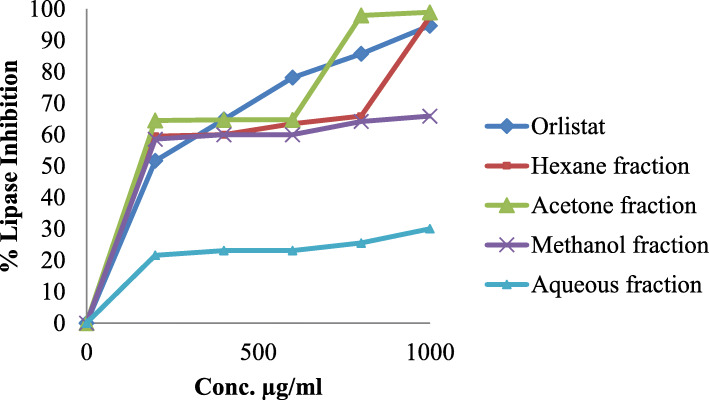
% Inhibition of lipase by standard orlistat and fractions from *R. rothschildianus* leaves

In this assay, the anti-obesity activity of fractions from *R. rothschildianus* leaves extract was compared to that of orlistat, a potent lipase inhibitory agent (Fig. 2 and Table 4). *R. rothschildianus* leaves were an excellent alternative natural source of lipase inhibitory agents. The acetone fraction showed an IC_50_ value of 26.3 ± 0.6 μg/ml, which was very close to that of the reference compound orlistat (12.3 ± 0.3 μg/ml). Hexane and methanol fractions only recorded moderate IC_50_ values, equal to 39.8 ± 0.3 μg/ml and 60.3 ± 0.4 μg/ml, respectively; while the aqueous fraction was inactive.

### α-Amylase inhibition activity

**Fig. 3:**
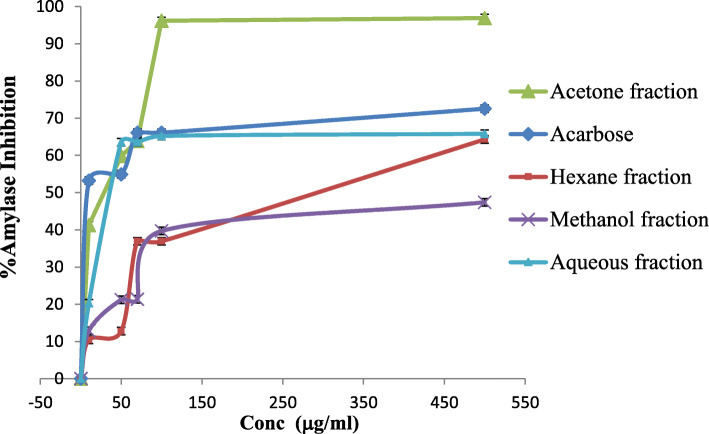
α-Amylase inhibition percentage of fractions from *R. rothschildianus* leaves compared to acarbose (standard compound)

In this assay, the anti-amylase activity of fractions from *R. rothschildianus* leaves extract was compared to that of acarbose, a potent α-amylase inhibitory agent (Fig. 3). The acetone fraction was the most potent inhibitor of α-amylase, with an IC_50_ of 19.0 ± 0.7 μg/ml, compared to 28.8 ± 1.2 μg/ml for acarbose, the reference compound. This suggested that *R. rothschildianus* might be a powerful herbal remedy for diabetes. The aqueous fraction only showed moderate activity in this assay with an IC_50_ value of 45.7 ± 0.3 μg/ml, while hexane had an IC_50_ value of 354.8 ± 1.2 μg/ml. The methanol fraction was inactive against the α-amylase enzyme. IC_50_ values were calculated for the four fractions (Table 4).

### α-Glucosidase inhibition activity

**Fig. 4:**
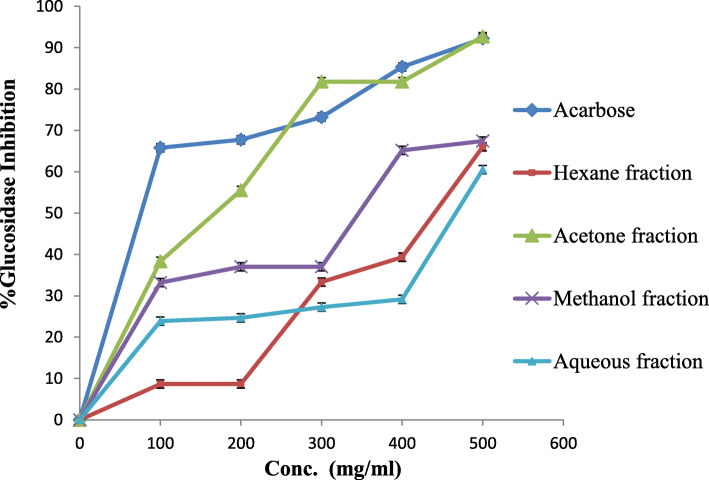
α-glucosidase inhibition percentage of fractions from *R. rothschildianus* leaves compared to acarbose

Results for α-glucosidase were compared with those for acarbose, a strong enzyme inhibitory agent, and IC_50_ values were calculated for the four fractions (Table 4 and Fig. 4). The acetone fraction exerted the greatest inhibitory action on α-glucosidase with an IC_50_ of 54.9 ± 0.3 μg/ml, compared with that of acarbose, the reference compound, at 37.1 ± 0.3 μg/ml. By contrast, the methanol extract fraction showed only moderate inhibition of α-glucosidase, with an IC_50_ of 251.2 ± 0.4 μg/ml, while the hexane and aqueous fractions were inactive in this assay.

### Anti-proliferative activity

**Fig. 5:**
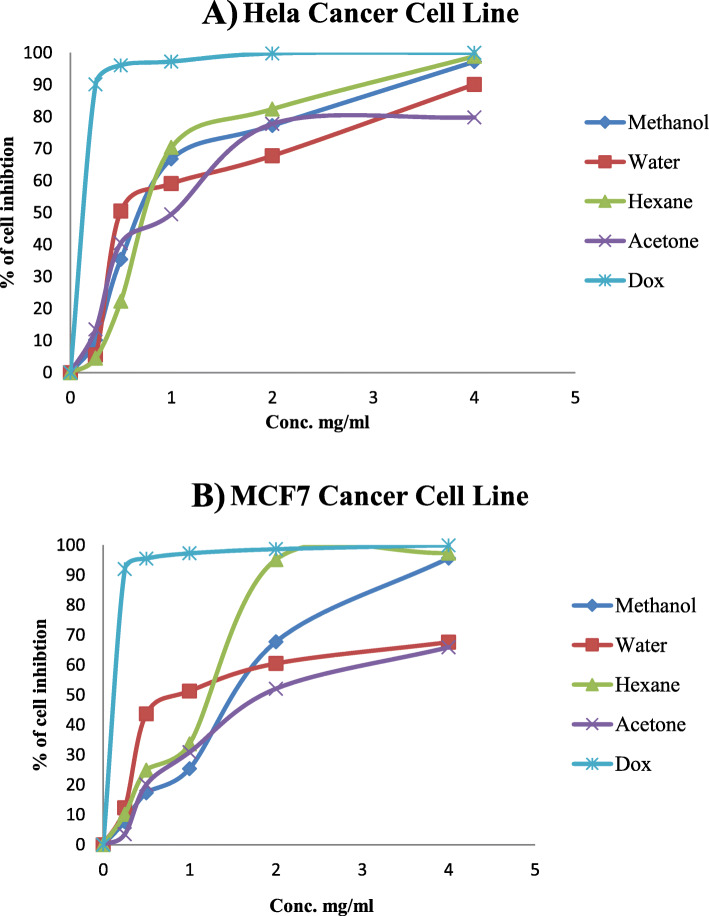
**a** HeLa Cancer cell line percentage inhibition by fractions from *R. rothschildianus* leaves, compared to the positive control (Dox). **b** MCF7 Cancer cell line percentage inhibition by fractions from *R. rothschildianus* leaves, compared to the positive control (Dox)

The results of treatment of HeLa and MCF7 cancer cells with five different concentrations in mg/ml for different extracts showed that the activity against the HeLa cancer cell line was better than against the MCF7 cancer cell line. However, hexane extract was the most potent extract at 4 mg/ml concentration with inhibition percentage 98.9 and 97.4% against HeLa and MCF7 cancer cell lines, respectively, while methanol extract at the same concentration showed potent activity against HeLa and MCF7 cancer cell lines with 97.2 and 95.6% inhibition percentage. HeLa cell percentage inhibition on exposure to fractions from *R. rothschildianus* leaves was documented, compared to the positive control doxorubicin (Fig. 5a). MCF7 cells percentage inhibition was similarly determined for the four fractions from *R. rothschildianus* leaves, compared to the control (Fig. 5b).

## Discussion

The DPPH radical scavenging assay is well known as a simple method for detecting antioxidant capacity in compounds. DPPH is a stable free radical that gives a purple color in alcohol solutions, and on reduction in the presence of hydrogen donating antioxidants, turns the solution colorless [29]. Therefore, *R. rothschildianus* leaves could be considered a natural source for antioxidants, especially for the acetone fraction, which showed an IC_50_ value of 6.3 ± 0.4 μg/ml. Similar results were also obtained for the hexane fraction, which had an IC_50_ value of 7.9 ± 1.3 μg/ml (Table 4). The results were compared to trolox, a potent antioxidant compound, with an IC_50_ equal to 3.1 ± 0.9 μg/ml. By contrast, the aqueous fraction only showed moderate antioxidant activity, with a higher IC_50_ value of 19.9 ± 0.7 μg/ml, while the methanol extract was inactive in this assay. These results were consistent with the presence of phenols and flavonoids in the plant, a powerful scavenger source for free radicals as shown with DPPH in this study. These results were in line with the diversity of phenolic compounds in plants, simple phenols like gallic acid, and more sophisticated phenolic acids like anthocyanins, hydroxyl cinnamic acid derivatives, and flavonoids. All these classes of compounds have received extensive attention due to their multiple physiological functions, especially free radical scavenging, anti-mutagenic, anti-inflammatory, and anti-carcinogenic activities [30]. As listed in Table 3, the acetone extract recorded the highest content of both phenolic compounds and flavonoids, 28.2 ± 0.8 mg of GAE/g and 107.3 ± 4.6 mg of RU/g, respectively. A previous report on some *Rumex* species found phenolic compounds in an ethanol extract from leaves of *Rumex vesicarius* L., which were possibly involved in free radical reactions, reducing the stable used DPPH radical to a yellowish colored diphenyl picrylhydrazine derivative from its original violet color [31].

*R. rothschildianus* leaves were an excellent alternative natural source of lipase inhibitory agents. The acetone fraction showed an IC_50_ value of 26.3 ± 0.6 μg/ml, which was very close to that of the reference compound orlistat (12.3 ± 0.3 μg/ml). Hexane and methanol fractions only recorded moderate IC_50_ values, equal to 39.8 ± 0.3 μg/ml and 60.3 ± 0.4 μg/ml, respectively; while the aqueous fraction was inactive. Pancreatic lipase is a major enzyme involved in enterocyte triglyceride absorption. Therefore, its inhibition represents an important strategy in the management of obesity [32]. Plants rich in phenolic compounds have been screened in several reports for anti-lipase activity. Lipase inhibitory activity ranging from 40 to > 70% has been found by in vitro tests in many different families, including Solanaceae (*Solanum̴ tuberosum*), Brassicaceae (*Brassica nigra* and *Raphanus sativus*), Rosaceae (*Malus domestica Borkh.* and *Filipendula ulmaria* (L.) Maxim.), Ericaceae (*Arctostaphylos uva-ursi* (L.) Spreng. and *Vaccinium myrtillus* L.), and Fabaceae (*Pisum sativum* L. and *Phaseolus vulgaris* L.) [33].

The acetone fraction was the most potent inhibitor of α-amylase, with an IC_50_ of 19.0 ± 0.7 μg/ml, compared to 28.8 ± 1.2 μg/ml for acarbose, the reference compound. This suggested that *R. rothschildianus* might be a powerful herbal remedy for diabetes. The aqueous fraction only showed moderate activity in this assay with an IC_50_ value of 45.7 ± 0.3 μg/ml, while hexane had an IC_50_ value of 354.8 ± 1.2 μg/ml. The methanol fraction was inactive against α-amylase. A possible explanation for the aqueous fraction being a good enzyme inhibitor was the presence of saponins. Earlier scientific investigations found that saponins were bioactive against diabetes [34]. The potent effect of the acetone extract fraction against amylase might be due to the high content of both phenolic compounds and flavonoids. *Corchorus olitorius* exerts α-amylase and α-glucosidase inhibitory effects due to constituents, especially phenolic compounds like caffeic acid [35].

The acetone fraction exerted the greatest inhibitory action on α-glucosidase with an IC_50_ of 54.9 ± 0.3 μg/ml, compared with that of acarbose, the reference compound, at 37.1 ± 0.3 μg/ml. By contrast, the methanol extract fraction showed only moderate inhibition of α-glucosidase, with an IC_50_ of 251.2 ± 0.4 μg/ml, while the hexane and aqueous fractions were inactive in this assay. The third category of oral hypoglycemic agents includes α-glucosidase inhibitors. There are a variety of α-glucosidase inhibitors, such as acarbose and voglibose, which usually are found in plant sources. They show valuable stabilization of blood glucose levels after a meal and have been used clinically in the management of diabetes mellitus [36, 37].

The results of treatment of both cancer cells (HeLa and MCF7) with various concentrations in mg/ml for different extracts showed that the general activity against the HeLa cancer cell line was better than MCF7 cancer cell line. The hexane extract showed potent anticancer activity at 4 mg/ml concentration with inhibition percentage 98.9 and 97.4% against HeLa and MCF7 cancer cell lines, respectively, while methanol extract at the same concentration showed potent activity against HeLa and MCF7 cancer cell lines with 97.2 and 95.6% inhibition percentage. From the previous results, the hexane fraction exerted a significant cytotoxic effect on both HeLa and MCF7 cell, with inhibition percentages reaching 99 and 92.4% at 4 mg/ml of hexane fraction concentration, respectively. This was consistent with the cytotoxic effects of both terpenoids and steroids, which were found in the hexane fraction. Diosgenin, a naturally occurring steroid and triterpenoids found in some plants, has been shown to inhibit breast cancer [38, 39].

## Conclusion

The results from this study on *R. rothschildianus* leaves indicated that the acetone extract fraction had significant potential in providing phytotherapies for diabetes and obesity, based on its potent inhibition of lipase, α-amylase, and α-glucosidase. In addition, the acetone fraction also showed a significant free radical scavenging activity. On the other hand, the hexane fraction showed significant inhibition of both HeLa and MCF7 cell lines, which might be related to its high content of terpenes and steroids. These observations in this study might lead to further in vivo studies to develop new natural pharmaceutical formulations effective in the treatment of obesity, diabetes mellitus, and some cancers.

## Data Availability

The datasets used and/or analyzed during the current study are available from the corresponding author on reasonable request.

## Abbreviations

PNPB: p-nitrophenyl butyrate
DNSA: 3,5-dinitrosalicylic acid
DMSO: Dimethyl sulfoxide
DPPH: 2,2-diphenyl-1-picrylhydrazyl
IC_50_: Half maximal inhibitory concentration
Trolox: 6-hydroxy-2,5,7,8-tetramethylchroman-2-carboxylic acid
A_B_: Absorbance of the blank solution
A_ts_: Absorbance of the tested sample solution
MCF7: Human breast cancer cell line
HeLa: Human cervix adenocarcinoma cell line
CAE: Catechin equivalent
GAE: Gallic acid equivalent
RUE: Rutin equivalent
*P*NPG: p-nitrophenyl glucopyranoside
Dox: Doxorubicin
Conc: Concentration
TPC: Total phenolic content
TCC: Total tannin content
TFC: Total flavonoid content
